# Reduction in young male suicide in Scotland

**DOI:** 10.1186/1471-2458-8-80

**Published:** 2008-02-29

**Authors:** Cameron Stark, Diane Stockton, Rob Henderson

**Affiliations:** 1 Centre for Rural Health, University of Aberdeen, The Greenhouse, Beechwood Business Park, Inverness IV2 3BL, UK; 2 Department of Public Health, NHS Highland, Assynt House, Beechwood Park, Inverness, IV2 3BW, UK; 3 Information Services Division, NHS National Services Scotland, Gyle Square, 1 South Gyle Crescent, Edinburgh EH12 9EB, UK; 4 Department of Public Health, NHS Grampian, Summerfield House, 2 Eday Road, Aberdeen AB15 6RE, UK

## Abstract

**Background:**

Rates of suicide and undetermined death increased rapidly in Scotland in the 1980's and 1990's. The largest increases were in men, with a marked increase in rates in younger age groups. This was associated with an increase in hanging as a method of suicide. National suicide prevention work has identified young men as a priority group. Routinely collected national information suggested a decrease in suicide rates in younger men at the beginning of the 21^st ^century. This study tested whether this was a significant change in trend, and whether it was associated with any change in hanging rates in young men.

**Methods:**

Joinpoint regression was used to estimate annual percentage changes in age-specific rates of suicide and undetermined intent death, and to identify times when the trends changed significantly. Rates of deaths by method in 15 – 29 year old males and females were also examined to assess whether there had been any significant changes in method use in this age group.

**Results:**

There was a 42% reduction in rates in 15 – 29 year old men, from 42.5/100,000 in 2000 to 24.5/100,000 in 2004. A joinpoint analysis confirmed that this was a significant change. There was also a significant change in trend in hanging in men in this age group, with a reduction in rates after 2000. No other male age group showed a significant change in trend over the period 1980 – 2004. There was a smaller reduction in suicide rates in women in the 15 – 29 year old age group, with a reduction in hanging from 2002.

**Conclusion:**

There has been a reduction in suicide rates in men aged 15 – 29 years, and this is associated with a significant reduction in deaths by hanging in this age group. It is not clear whether this is related to a change in method preference, or an overall reduction in suicidal behaviour, and review of self-harm data will be required to investigate this further.

## Background

Rates of suicide and deaths of undetermined intent increased markedly in Scotland in the last two decades of the twentieth century. [1-3] This was driven by high rates of suicide and undetermined intent deaths among men. The combined all age suicide and undetermined death rate among men increased by 35% between the period 1981–1985 and 1996–1999. During this time, there was a 7% decrease in the female all age rate. However, there were age group differences within genders. For example, between the periods 1981–1985 and 1996–1999 the combined suicide and undetermined death rate among men aged 15–24 years and 25–34 years increased by 97% and 86% respectively. [4] As a result of these increases, young men were identified as a priority group in the Scottish suicide prevention strategy. [5]

While suicide rates increased in both deprived and non-deprived groups, the greatest increase occurred among men living in the most deprived areas of the country. This resulted in a widening of social disparities in male suicide in Scotland. [6]

Methods of suicide in Scotland changed during the last two decades of the 20^th ^Century. During the early 1980's, hanging, strangulation and suffocation had a similar rate to self-poisoning, but by 1996 – 9 hanging was the commonest method of male suicide in Scotland, having increased by 96% compared to 1981 – 5. Poisoning with gases, principally car exhausts, increased in men in the 1980's but reached a plateau before decreasing in the 1990's, coinciding with the introduction of catalytic converters [4]

Reductions in young male suicide rates have been reported in the United States [7] and Australia. [8] We noted a decrease in Scottish all age male suicide rates in routinely published data in 2003 and 2004 compared to previous years. Review of age-specific rates suggested that this was related to a decrease in suicide rates in younger men. We set out to find if this change in trend was statistically significant. As the rise in male suicide rates was associated with an increase in hanging, we hypothesised that there might have been a move away from hanging as a method of suicide in younger men.

## Methods

The General Register Office for Scotland (GROS) provided anonymised routinely collected information on deaths by suicide and undetermined intent for 1980 – 2004 (ICD-9: E950-E959 and E980-E989 respectively; ICD 10: X60-X84 and Y10-Y34 respectively). This included age, gender and method of suicide as recorded on the death certificate. We calculated rates by age and gender using population denominators provided by the GROS.

Joinpoint regression analysis (version 3) was used to assess changes in trend in the age-specific suicide rates, and in method in younger men. [9] This method tests whether an apparent change in trend is statistically significant. Years in which trends change are termed joinpoints. [10] The log linear option was used which allows analysis of the estimated annual percentage change in rate (EAPC). The test of annual percentage change uses an asymptotic t-test and a permutation test to determine the number of joinpoints. The constant variance option was used. Monte Carlo simulation is used to calculate p-values for a series of permutation tests. Multiple tests are performed, and the significance level is adjusted by the programme to take account of these multiple comparisons. A division in to two segments by a joinpoint reduces the number of observations in the sections, and so EAPCs for each segment may not be significant. The EAPCs are based on the asymptotic t-test, while the permutation test determines the number of joinpoints. The permutation test is a more powerful method of determining the overall trend for the segments.

## Results

### Age-specific rates by Gender

**Figure 1:**
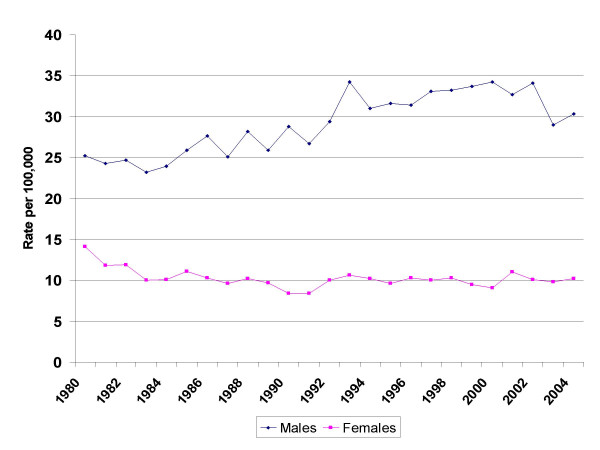
Suicide and undetermined deaths in Scotland 1980 – 2004. All age rates by gender.

There was a marked reduction in 15 – 29 year old men, from 42.5/100,000 in 2000 to 24.5 in 2004, a decrease of 42%. The overall reduction in male rates (Figure 1) was a significant change in trend (Table 1), but within the age groups examined, in males only the 15 – 29 year old group had a similar significant change in trend in the same time period. The increase in EAPC in 15 – 29 year old men had slowed in 1992 – 2000, but showed a marked decrease from 2000 onwards. There were no significant changes in trend in any other male age group in the period reviewed.

**Table 1:** Trends in Age-Specific Suicide Rates in Scotland 1980 – 2004

**Males**				**Females**			
**Joinpoints (Years)**	**Time Period**	**EAPC^1^**	**95% CI^2^**	**Joinpoints (Years)**	**Time Period**	**EAPC^1^**	**95% CI^2^**

**All Ages**							
1 joinpoint	1980 – 2000	+1.97	+1.5 – +2.4	1 joinpoint	1980 – 1983	-10.6	-19.8 – -0.4
2000	2000 – 2004	-3.67	-8.4 – +1.3	1983	1983 – 2004	-0.01	-0.56 – +0.5
							
**15 – 29**							
3 joinpoints	1980 – 1983	-4.7	-16.2 – +8.3	1 joinpoint	1980 – 1996	5.56	+3.6 – +7.6
1983	1983 – 1992	+8.4	+5.4 – +11.5	1996	1996 – 2004	-2.22	-7.4 – +3.2
1992	1992 – 2000	+2.9	-0.5 – +6.5				
2000	2000 – 2004	-11.5	-18.4 – -4				
							
**30 – 44**							
0 joinpoints	1980 – 2004	+2.42	+2.0 – +2.8	1 joinpoint	1980 – 1990	-2.7	-5.6 – +0.3
				1990	1990 – 2004	2.83	+0.9 – +4.7
							
**45 – 59**							
0 joinpoints	1980 – 2004	-0.3	-0.7 – +0.2	1 joinpoint	1980 – 1988	-9.2	-12.7 – -5.5
				1988	1988 – 2004	+0.8	-0.6 – +2.2
							
**60 and over**							
0 joinpoints	1980 – 2004	-1.0	-1.5 – -0.6	0 joinpoints	1980 – 2004	-3.0	-3.72 – -2.3

^1 ^Estimated annual percentage change in rate

^2 ^95% confidence interval

In 15 – 29 year old women a significant change in trend was found in 1996 with a considerably smaller EAPC than the same male age group. The oldest female age group showed a steady reduction over the time period, while the two intermediate groups had a small positive EAPC from the late 1980's/early 1990's.

In view of the rapid decrease in young male rates in younger men, the methods of suicide in this age group were examined in more detail to test the hypothesis that the decrease in male death rates in this age group may have been associated with a decrease in the use of hanging as a method of suicide.

### Method changes

**Figure 2:**
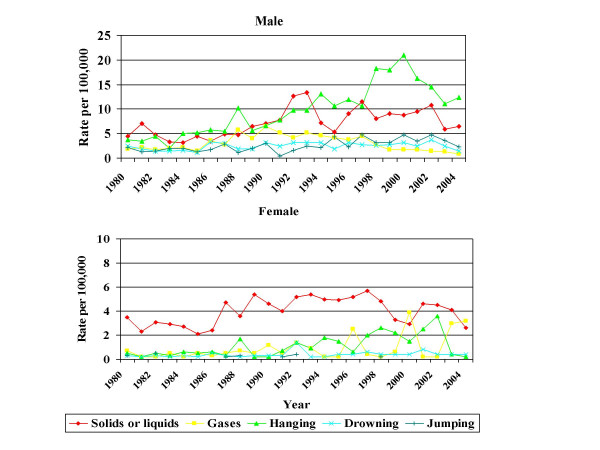
Suicide and undetermined deaths in Scotland 1980 – 2004. Methods in 15 – 29 year old age group by gender.

Methods in 15 – 29 year old men are shown in Figure 2. Table 2 displays the results of a joinpoint analysis of method trend in this age group. Only poisoning, hanging and use of gases showed any change in trend over the time period. The change in trend in hanging was at 2000, while the changes in trend in poisoning and gases occurred earlier in the 1990s.

**Table 2:** Trends in Methods of Suicide and Undetermined Intent Death in 15 – 29 year old males in Scotland, 1980 – 2004

**Joinpoints (Years)**	**Time Period**	**EAPC^1^**	**95% CI^2^**
**Poisoning**			
2 joinpoints	1980 – 1984	-14.3	-32.5 – +8.9
1984	1984 – 1992	+15.1	+4.0 – +27.4
1992	1992 – 2004	-2	-6.4 – +2.4
			
**Gases**			
1 joinpoint	1980 – 1993	+10.3	+6.4 – +15.2
1993	1993 – 2004	-15.3	-19.6 – -10.8
			
**Hanging**			
1 joinpoint	1980 – 2000	+9.4	+7.4 – +11.5
2000	2000 – 2004	-11.9	-29.1 – +9.4
			
**Drowning**			
0 joinpoints	1980 – 2004	+2.0	+0.19 – +3.8
			
**Firearms**			
0 joinpoints	1980 – 2004	-4.7	-7.3 – -2.1
			
**Cutting**			
0 joinpoints	1980 – 2004	+0.88	-3.8 – +5.8
			
**Jumping**			
0 joinpoints	1980 – 2004	+4.3	+1.7 – +6.9

^1 ^Estimated annual percentage change in rate

^2 ^95% confidence interval

Joinpoints were also calculated for this age group in women. It was not possible to calculate joinpoints for all methods, as the dependent variable cannot be zero in a joinpoint analysis. Poisoning in 15 – 29 year old women had one joinpoint in 1994 (1980 – 1994, EAPC +5.8, 95% CI +2.5 – +9.2, 1994 – 2004, EAPC -4.8, 95% CI -9.7 – +0.4). Hanging had one joinpoint in 2002 (1980 – 2002, EAPC +10.4, 95% CI +5.9 – +15.2, 2002 – 2004 EAPC -74, 95% CI-95.6 – +49.2).

## Discussion

The decrease in male rates was attributable to a decrease in rates in 15 – 29 year old men. Further review identified that this was caused by a decrease in hanging deaths, supporting the hypothesis that there had been a method change in younger men. There was evidence of a more modest reduction in overall rates in 15 – 29 year old women which pre-dated the male decrease. There had, however, been a recent decrease in hanging deaths in young women, although this was based on a far lower number of deaths.

Most deaths by hanging in an study in England were in community settings, and therefore not amenable to restriction of method. [11] The final number of deaths by suicide in an area is a product of the frequency of attempted suicide, and the case-fatality rate of the method chosen. A method such as self-poisoning has a lower case-fatality rate than methods such as hanging and use of firearms. [12] As hanging is a method of self-harm with a high case-fatality rate, a change in death rates could occur by an alteration in rates of hanging alone. Changes in method preference have been suggested as a possible cause for the increase in male suicide rates in Scotland in the 1990's. [4] If younger age groups move away from hanging as a method of self-harm, then a reduction in death rates could occur with no underlying change in the frequency of self-harming behaviour. The lack of any increase in deaths from other methods in young men suggests a true decrease in suicidal behaviour, but careful review of non-fatal self-harm statistics in Scotland will be required to test the hypothesis that there has been a decrease in self-harm in this age group, rather than a change in method preference. There is no apparent reason why there should have been a differential effect on rates of death by hanging, and this requires further exploration.

Reasons for a change in method choice in younger people, or a reduction in suicidal behaviour, are not clear. The factors underlying method choice in suicide are poorly understood. [12] Morrell *et al* hypothesise that hanging may be more sensitive to population interventions than suicide methods which they consider as more impulsive, and less likely to involve planning. [8] Gunnell et al also note the theoretical importance of population level interventions in methods such as hanging, where limitation of population method availability is not possible. [12]

Several English speaking countries have reported decreases in suicide in younger men, including America [7] and Australia. [8] In the United States, suicides involving firearms decreased in adolescents, although there was an increase in hanging deaths in white males. In Australia, Morrell *et al*report a large decline in hanging deaths which largely accounted for the observed decrease in suicide rates in 20 – 24 year old men. [8] The authors relate the reduction to the Australian National Youth Suicide Prevention Strategy, a programme involving primary prevention programmes, capacity building in services and treatment support.

It is possible that a recent government focus on excluded groups, and on mental health and well-being, has had an impact. Scotland is part of the United Kingdom (UK), a political union which includes England, Wales and Northern Ireland, although Scotland has an independent legal system. Macroeconomic and taxation policy are set by the UK Parliament. A devolved Scottish parliament was established in 1999 for the first time since 1707. [13], The parliament has responsibilities for specific areas including health and education policy. This has resulted in gradual differences emerging between Scotland and its neighbours. Tackling social exclusion was already accorded a high profile prior to devolution [14] and this has been maintained by policy initiatives from the Scottish Government which has continued to focus on social exclusion, including work targeted on deprived communities. [15] There has been a substantial focus of work on programmes aimed at people with mental ill-health, who are viewed as an excluded group. Policy and practice to promote positive mental health and prevent mental illness is coordinated by the National Programme for Improving Mental Health and Wellbeing. [5] Since 2001, the National Programme has taken forward work including '*Choose Life*' (the Scottish suicide prevention strategy), [16] the '*See Me*' anti-stigma programme, [17] '*Breathing Space*' (a confidential telephone helpline targeted at young men), [18] and the development of a recovery-oriented mental health programme, the Scottish Recovery Network. [19]

It is not possible to link these initiatives confidently to the reduction in suicide in young people. There has been, however, considerable media attention on mental health and suicide in Scotland, and the introduction of the *Breathing Space *helpline, the *Choose Life *programme, anti-stigma work and a large investment in media publicity may have contributed to the reductions seen in 2002 and subsequent years. The Choose Life project team have introduced media guidelines on the coverage of suicide, which include advice against the reporting of suicide method. The beginning of the reduction in young men seems to predate the likely impact of these national initiatives, but further exploration of the impact of these policies is essential.

There was no significant change in trends in older men, or any recent change in trends in women. Much of the media publicity on suicide in Scotland has focused on suicide among younger men (Nicola Hughes, Choose Life Communications Manager. Personal Communication, 2006). It may be that work including help-lines, encouraging help-seeking behaviour and challenging stigma was interpreted by older men as referring to a younger age group, and women may not see it as relating to them at all. Views of mental ill-health, stress and help-seeking may be more ingrained in older men, leading to delays in the impact of initiatives which seek to address them. It is notable however, that this is the group of men who had shown large increases in suicide rates in previous decades, raising the possibility of a cohort effect. [4] Cohort effects are where a particular group of people, in this case a birth cohort, carry a risk with them through the life cycle. Disentangling age, period and cohort effects in male suicide in Scotland may offer helpful insights into the factors contributing to suicidal behaviour among older men, although the speed of the decrease in younger men suggests that a cohort effect is less likely to be a possible explanation in this age group.

## Conclusion

Suicide among young men in Scotland has decreased, and this is associated with a reduction in hanging deaths. Although deaths from gases and from poisoning have also decreased in this age group of men, the temporal association with the overall reduction in male deaths is with the reduction in hanging. It is not possible to tell from suicide data alone if this is related to a decrease in suicidal attempts, or to a shift to a method with a lower case fatality rate. The lack of any increase in trends in other methods of suicide in this age group suggests that this may be a true decrease in suicidal behaviour, but this will require further investigation using information on hospital treated self-harm.

It is tempting to associate the reduced suicide rate in younger men, and to an extent in younger women, with population level initiatives to encourage help-seeking, reduce stigma, increase social support and target suicidal behaviour. Further review of the impact of the national strategies will be required before any definite link can be drawn. The lack of any change in trend in older men and in women suggests that review of these national efforts would be appropriate in relation to these groups.

## Competing interests

The author(s) declare that they have no competing interests.

## Authors' contributions

CS contributed to the study design, data analysis and writing of the paper. DS contributed to the data analysis and the writing of the paper. RH contributed to the study design and the writing of the paper. All authors read and approved the final manuscript.

## Pre-publication history

The pre-publication history for this paper can be accessed here:



## References

[B1] McLoone P, Crombie IK (1987). Trends in suicide in Scotland 1974–84: an increasing problem. BMJ.

[B2] Crombie IK (1990). Suicide in England and Wales: an examination of divergent trends. British Journal of Psychiatry.

[B3] Stark C, Matthewson F (2000). Differences in suicide rates may be even more pronounced. BMJ.

[B4] Stark C, Hopkins H, Gibbs D, Rapson T, Belbin A, Hay A (2004). Trends in suicide in Scotland 1981 – 1999: age, method and geography. BMC Public Health.

[B5] Scottish Executive (2003). National Programme for Improving Mental Health and Wellbeing Action Plan 2003 – 6.

[B6] Boyle P, Exeter D, Feng Z, Flowerdew R (2005). Suicide gap among young adults in Scotland: population study. BMJ.

[B7] Bridge JA, Barbe RP, Brent DA (2005). Recent trends in suicide among US adolescent males, 1992 – 2001. Psychiatric Services.

[B8] Morrell S, Page AN, Taylor RJ (2007). The decline in Australian young male suicide. Social Science and Medicine.

[B9] National Cancer Institute (2005). Joinpoint Regression Program Version 3.0. http://srab.cancer.gov/joinpoint.

[B10] Kim HJ, Fay MP, Feuer EJ, Midthune DN (2000). Permutation tests for joinpoint regression with application to cancer rates. Statistics in Medicine.

[B11] Bennewith O, Gunnell D, Kapur N, Turnbull P, Simkin S, Sutton L, Hawton K (2005). Suicide by hanging: a multicentre study based on coroners' records in England. British Journal of Psychiatry.

[B12] Gunnell D, Bennewith O, Hawton K, Simkin S, Kapur N (2005). The epidemiology and prevention of suicide by hanging: a systematic review. International Journal of Epidemiology.

[B13] Public Information Service (2006). How the Scottish Parliament Works.

[B14] Scottish Office (1998). Social Exclusion – A Consultation Paper.

[B15] Scottish Executive (1998). Social Inclusion: Opening the door to a better Scotland.

[B16] Scottish Executive (2002). Choose Life A National Strategy and Action Plan to Prevent Suicide in Scotland.

[B17] http://www.seemescotland.org.uk/index.php.

[B18] http://www.breathingspacescotland.co.uk/bspace/displayhomefocus.jsp;jsessionid=A3A2E211E619646A4DD5FFEF838666D4?p_applic=CCC&p_service=Content.show&pContentID=252&.

[B19] http://www.scottishrecovery.net/content/.

